# Effects of Sex Differences on Conditioned Fear Extinction and Safety Learning in C57BL/6J Mice

**DOI:** 10.3390/brainsci16030336

**Published:** 2026-03-21

**Authors:** Zhuoqun Liu, Haoxuan Pan, Huimeng Lei, Xiaohong Sun

**Affiliations:** School of Basic Medical Sciences, Beijing Key Laboratory of Neural Regeneration and Repair, Key Laboratory for Neurodegenerative Diseases of the Ministry of Education, Laboratory for Clinical Medicine, Capital Medical University, Beijing 100069, China; zq_liu@mail.ccmu.edu.cn (Z.L.); phx@mail.ccmu.edu.cn (H.P.); asd8195162@ccmu.edu.cn (X.S.)

**Keywords:** sex differences, conditioned fear, fear extinction, fear retrieval, safety conditioning, freezing behavior

## Abstract

**Objectives:** Females are often underrepresented in preclinical fear research due to concerns over estrous cycle related variability. This study examined whether there were differences between female and male C57BL/6J mice in terms of fear extinction and safety learning, aiming to verify the inclusion of both sexes in fear regulation research. **Methods:** Mice underwent a 5-day fear conditioning and extinction protocol, with recent (Day 6) and remote (Day 13) retrieval tests. A separate cohort received unpaired tone-shock safety conditioning over two days, followed by recent and remote retrieval. Freezing percentage and locomotor distance, among other measures, were quantified to compare behavioral responses between sexes. **Results:** During fear acquisition and extinction, females and males showed comparable conditioned fear and progressive extinction, with no sex differences in freezing percentage, bout counts, or locomotor distance. Freezing remained low during both recent and remote retrieval in both sexes. In the safety-conditioning task, the safety cue reduced freezing relative to contextual baseline, contextual freezing declined from recent to remote retrieval, and no sex differences were observed across measures. **Conclusions:** Female and male C57BL/6J mice exhibit equivalent performance in auditory fear conditioning, extinction, retrieval, and safety learning under matched conditions. These findings support equitable inclusion of both sexes in preclinical fear-regulation studies, enhancing translational relevance without added behavioral variability.

## 1. Introduction

Post-traumatic stress disorder (PTSD) is a disease related to fear. Its characteristic is the impairment of fear regulation function, and patients will exhibit persistent or excessive fear responses even in a safe situation [1,2]. The main pathological feature of PTSD is the inability to suppress or eliminate traumatic fear memories. The key to successfully adapting to a dynamic environment lies in the ability to distinguish between signals that indicate threats and those that indicate safety, thereby enabling one to make decisions regarding avoidance and exploration based on specific circumstances. The ability to continuously assess the value of cues depends on cognitive flexibility. When this process is disrupted, there will be a tendency towards excessive generalized fear, which in turn leads to the emergence of negative behaviors. This is a typical symptom of stress and anxiety disorders (including PTSD) [3,4,5,6,7,8]. Therefore, the lack of cognitive flexibility and the improper suppression of fear expression are regarded as the core behavioral characteristics of PTSD [5,9,10,11].

The Pavlovian method of fear conditioning experiments has been widely used to elucidate the neural and behavioral mechanisms of fear regulation. In this model, a neutral conditioned stimulus (CS) is paired with an aversive unconditioned stimulus (US), eliciting a conditioned fear response. In the subsequent extinction training, the conditioned stimulus is repeatedly presented while the unconditional stimulus is no longer presented. This behavioral paradigm can quantify fear inhibition and has become the cornerstone of basic research and translational research on fear regulation [12,13,14,15]. In addition to extinction, safety learning provides a complementary measure of fear regulation and has been linked to PTSD symptom severity. Within this framework, safety learning represents a specific phenomenon of conditioned inhibition. A signal indicating that no aversive event will occur will suppress the fear response [16,17]. The important point is that these patterns share significant similarities with exposure therapy at both the conceptual and operational levels. And exposure therapy is currently the most effective clinical method for treating PTSD [18,19,20], providing a high level of surface validity for the simulation treatment mechanism [15].

Despite decades of research on fear conditioning, it is still unclear whether the inclusion of female subjects has introduced significant variable factors that might interfere with the research results. This concern has led to the exclusion of female subjects from the study. Sex differences are an important biological variable used to model mental disorders, as there are often differences between men and women in terms of susceptibility, symptom manifestation, and treatment response [21,22,23,24]. PTSD is a mental disorder with a more pronounced gender difference. Although the recovery rates are similar, the lifetime prevalence rate of the disorder is significantly higher in women than in men [22,23,25,26,27,28,29]. However, most of the preclinical studies on post-traumatic stress disorder have only used male animals as experimental subjects [22,23,30,31]. This bias is largely due to an assumption that hormonal fluctuations during the estrus cycle increase the variability of behavior [32,33]. However, an increasing amount of evidence has challenged this notion. These studies have emphasized the importance of including women in behavioral neuroscience research in order to improve generalizability and translational validity [23,32,33,34,35,36].

In the present study, both female and male C57BL/6J wild-type mice were tested using classical auditory fear-conditioning and safety-learning paradigms. By comparing the freezing behavior and locomotor activity of male and female mice during the acquisition, extinction, and reacquisition phases of fear conditioning, as well as during the safety-conditioning stage, this study aims to assess the feasibility and reliability of incorporating female mice into investigations of conditioned fear extinction and safety learning. We hypothesized that, under carefully controlled experimental conditions, female and male mice would exhibit comparable fear acquisition, extinction, and safety learning, supporting the validity of including both sexes in future studies. Furthermore, this work highlights the importance and applicability of incorporating sex-specific analyses in investigations of fear regulation.

## 2. Materials and Methods

### 2.1. Animals

All experiments were conducted according to protocols approved by Animal Experiments and Experimental Animal Welfare Committee of Capital Medical University (Approval ID: AEEI-2025-683). Specific pathogen-free C57BL/6J wild-type mice (females and males, 2–3 months old, weighing 20–30 g) were obtained from Beijing Vital River Laboratory Animal Technology Co., Ltd., Beijing, China. A total of 45 mice were used in this study, including 27 mice in the fear conditioning cohort (13 males and 14 females) and 18 mice in the safety-conditioning cohort (9 males and 9 females). All mice were individually housed in the barrier facility of the Laboratory Animal Center at Capital Medical University under standard laboratory conditions. All procedures were performed in accordance with the ARRIVE guidelines and efforts were made to minimize suffering, including immediate monitoring of pain-related behaviors and early euthanasia criteria if necessary. Mice were randomly assigned to experimental cohorts using a random number table prior to behavioral testing. The order of behavioral testing was arranged in a randomized or pseudo-randomized manner across animals to minimize potential order effects.

### 2.2. Behavioral Tasks

#### 2.2.1. Apparatus

Behavioral testing was conducted using coded animal identifiers to reduce potential observer-related bias. Behavioral experiments were conducted using a conditioned fear system, behavioral conditioning controller, and the VisuTrack rodent behavior analysis platform V2.0 (Shanghai Xinruan Information Technology Co., Ltd., Shanghai, China). Behavioral stimulus delivery and freezing quantification were performed automatically using the VisuTrack rodent behavior analysis system.

#### 2.2.2. Fear Conditioning

All conditioning, extinction and retrieval procedures were performed in the behavioral conditioning controller (Shanghai Xinruan). To habituate animals to the testing environment, each mouse was placed in the experimental chamber for 10 min on the day prior to training.

On fear conditioning day (Day 1), mice underwent a 3 min baseline period followed by five tone–shock pairings (CS: 30 s, 80 dB, 8 kHz tone; US: 2 s, 0.7 mA footshock). The tone and shock co-terminated, and the intertrial interval (ITI) was 60 s.

During fear extinction days (Days 2–5), mice were placed in a context distinct from the conditioning environment after a 3 min baseline and received twelve tone-alone trials (CS: 30 s, 80 dB, 8 kHz tone; ITI: 60 s). The number of extinction sessions was chosen to provide a sufficiently robust but spaced extinction regimen before recent and remote retrieval testing. Specifically, mice received 12 tone-alone trials per day across 4 consecutive days (48 trials total), a design comparable to prior mouse auditory fear-extinction studies using multi-day extinction protocols with approximately 36–50 total unreinforced CS presentations. A spaced, multi-day schedule was selected to permit both within-session extinction and between-session consolidation before retention testing [37,38]. Relative to the fear-conditioning context, the extinction context differed in wall and floor color, the presence of 15 dB white noise, and chamber cleaning with 4% acetic acid rather than 70% ethanol, thereby establishing a multimodal context distinguishable from that used during fear learning. The extinction context was intentionally modified to emphasize auditory fear extinction and retrieval while minimizing contextual freezing.

On fear retrieval days (Day 6 and Day 13), mice were placed in the same environment used for extinction training. Following a 3 min baseline, each mouse received four tone-alone trials (CS: 30 s, 80 dB, 8 kHz tone; ITI: 60 s) to assess retention of fear memory. Stimulus delivery and automated freezing analysis were controlled by the VisuTrack rodent behavior analysis system (Shanghai Xinruan Information Technology Co., Ltd.).

#### 2.2.3. Safety Conditioning

Both male and female mice underwent one day of habituation in the safety-conditioning context. During habituation, each mouse received five neutral cue presentations (CS: 30 s, 4 kHz, 100 dB; ITI: 120 s) and then 10 min of free exploration to reduce novelty-induced fear. During habituation, mice were exposed to a neutral auditory stimulus (4 kHz, 100 dB) that differed from the safety-conditioning cue (8 kHz, 80 dB). This was done to familiarize animals with the occurrence of auditory stimuli in the testing context while avoiding prior nonreinforced exposure to the exact safety cue used later during conditioning and retrieval, which could have reduced its associability via latent inhibition [39,40,41].

On the first day of safety conditioning, following a 3 min baseline, mice received five CS presentations (CS: 30 s, 80 dB, 8 kHz tone) explicitly unpaired with five foot shock US (US: 2 s, 0.7 mA). The CS–US intervals varied pseudo-randomly between 40–60 s, ensuring that the US never occurred during or immediately after a CS. The average CS–US interval was 50 s, and ITIs were 60–120 s. After 24 h, animals underwent a second conditioning session identical to Day 1.

All mice were tested 24 h after the second conditioning session for recent retrieval, and again 18 days later for remote retrieval, both conducted in the original safety-conditioning context. Each retrieval session began with a 3 min baseline followed by five CS trials (CS: 30 s, 80 dB, 8 kHz; ITI: 60–120 s). Stimulus programming and freezing quantification were managed automatically using the VisuTrack behavioral analysis system (Shanghai Xinruan).

### 2.3. Statistical Analysis

All statistical analyses and graphing were performed using GraphPad Prism 9 (GraphPad Software, San Diego, CA, USA). Data that followed a normal distribution and showed homogeneity of variance are expressed as mean ± standard error of the mean (SEM).

Two-way repeated-measures ANOVA was applied to compare freezing percentages across groups, while within-group changes across time points were analyzed using repeated-measures (rm) ANOVA. A *p* value < 0.05 was considered statistically significant. When significant main effects were observed, multiple comparisons were performed for further analysis. For ANOVA results, F statistics, degrees of freedom, exact *p* values, and partial eta squared (ηp^2^) are reported. For post hoc comparisons, adjusted *p* values and effect sizes (Hedges’ g) are provided where appropriate. When manual data inspection or exclusion was required, the analysis was performed using coded datasets so that the investigator was blind to group allocation until completion of the primary analysis.

Sample Size Considerations. No formal a priori power calculation was performed for the present study. Sample sizes were determined with reference to previously published mouse auditory fear-conditioning and safety-learning studies using comparable paradigms, together with our laboratory’s prior experience with these behavioral procedures and consideration of animal-use reduction principles. Under these conditions, the selected sample sizes were considered sufficient to detect robust learning-related behavioral changes, although they may have been less sensitive for detecting subtle sex effects or sex-by-time interactions.

## 3. Results

### 3.1. Fear Conditioning, Extinction, and Retrieval Task

**Figure 1:**
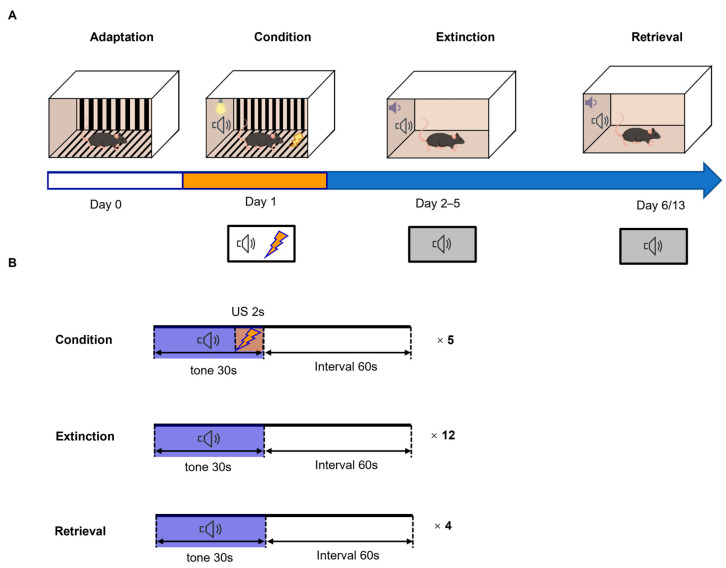
Schematic for the fear conditioning, extinction, and retrieval procedure. (**A**) Experimental context and procedure. (**B**) Timing of conditioned stimulus presentation and intertrial interval.

A previously established conditioning, extinction, and retrieval protocol was applied (Figure 1A,B), and freezing during the CS was quantified to assess CS-evoked responses.

#### 3.1.1. Sex Does Not Significantly Affect Fear Levels in C57BL/6J Wild-Type Mice During Conditioned Fear Acquisition, Extinction, or Retrieval

**Figure 2:**
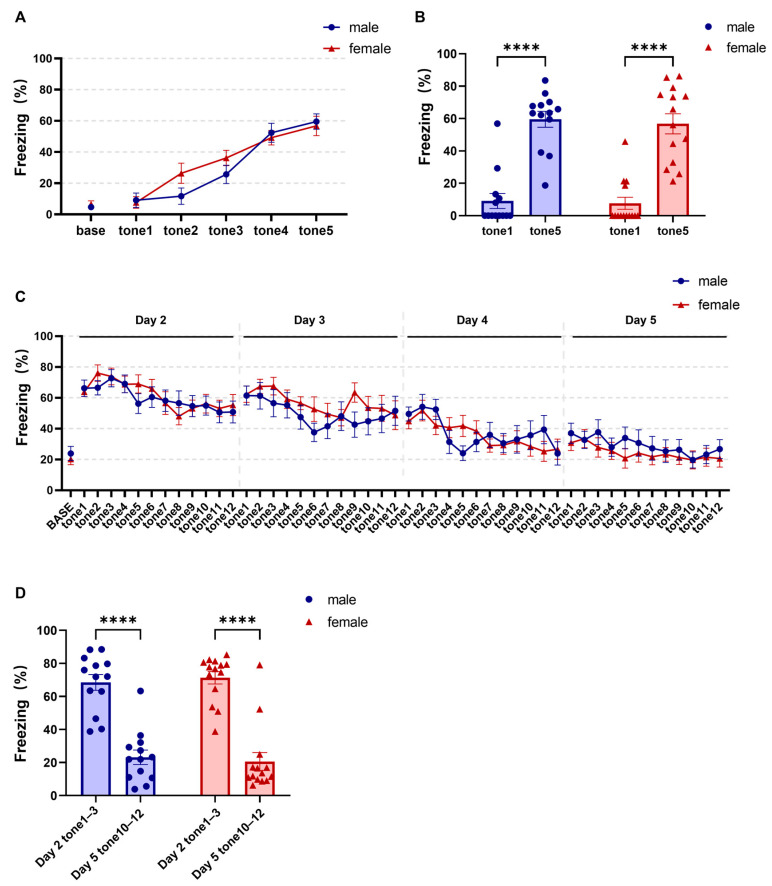
Comparable conditioned fear acquisition, and extinction in female and male mice. (**A**) Freezing across tones 1–5 during fear conditioning. (**B**) Freezing during tone 5 versus tone 1 in both sexes. (**C**) Freezing across fear extinction. (**D**) Freezing during early extinction (tones 1–3, Day 2) versus late extinction (tones 10–12, Day 5) in both sexes. Data are presented as mean ± SEM; *n* = 13 males and 14 females. Asterisks indicate statistically significant comparisons. Exact *p* values, test statistics, and effect sizes for the comparisons shown in this figure are reported in Section 3.

During fear conditioning, freezing did not increase before tone onset or during the first tone, but rose markedly by the fifth tone as the CS–footshock pairings proceeded, indicating successful acquisition of conditioned fear in both sexes. No significant sex differences were observed during fear conditioning (Figure 2A,B, A two-way repeated-measures ANOVA, main effect of trial: F (3.567, 89.17) = 62.19, *p* < 0.0001, ηp^2^ = 0.713; main effect of sex: F (1, 25) = 0.4737, *p* = 0.498, ηp^2^ = 0.019; sex × trial interaction: F (5, 125) = 1.855, *p* = 0.107, ηp^2^ = 0.059; post hoc comparisons, male tone1 vs. tone5 adjusted *p* < 0.0001, Hedges’ g = 2.13; female tone1 vs. tone5 adjusted *p* < 0.0001, Hedges’ g = 2.14; males = 13, females = 14).

During fear extinction, CS-evoked freezing gradually decreased across trials, with no significant sex effects (Figure 2C; two-way repeated-measures ANOVA, main effect of extinction trial: F (8.606, 215.2) = 15.60, *p* < 0.0001, ηp^2^ = 0.384; main effect of sex: F (1, 25) = 0.019, *p* = 0.890, ηp^2^ = 0.001; sex × extinction trial interaction: F (48, 1200) = 0.982, *p* = 0.509, ηp^2^ = 0.038; males = 13, females = 14). Freezing during tones (1–3) on the first extinction day was significantly higher than during tones (10–12) on the final extinction day. However, males and females did not differ at either time point, indicating robust extinction in both sexes (Figure 2D; two-way repeated-measures ANOVA, main effect of extinction phase [Day 2 tones 1–3 vs. Day 5 tones 10–12]: F (1, 25) = 141.9, *p* < 0.0001, ηp^2^ = 0.580; main effect of sex: F (1, 25) = 0.0006, *p* = 0.892, ηp^2^ < 0.001; sex × extinction-phase interaction: F (1, 25) = 0.446, *p* = 0.510, ηp^2^ = 0.018; post hoc comparisons, male Day 2 tones 1–3 vs. Day 5 tones 10–12, adjusted *p* < 0.0001, Hedges’ g = 2.02; female Day 2 tones 1–3 vs. Day 5 tones 10–12, adjusted *p* < 0.0001, Hedges’ g = 2.28; males = 13, females = 14).

**Figure 3:**
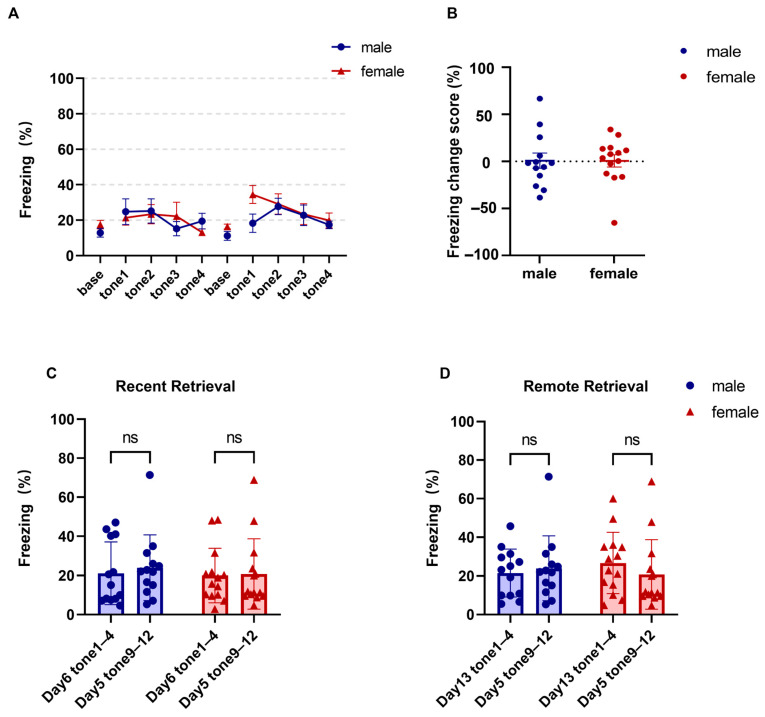
Comparable conditioned fear extinction, and retrieval in female and male mice. (**A**) Freezing during fear retrieval. (**B**) Change in freezing from late extinction to Tone 1 of remote retrieval in female and male mice. Each point represents one animal. Positive values indicate higher freezing during the first tone of remote retrieval than during late extinction, whereas negative values indicate no rebound or lower freezing relative to late extinction. (**C**) Freezing during recent retrieval versus the final day of extinction. (**D**) Freezing during remote retrieval versus the final day of extinction. Data are presented as mean ± SEM; *n* = 13 males and 14 females. Exact *p* values, test statistics, and effect sizes for the comparisons shown in this figure are reported in the Section 3.

During fear retrieval, CS-evoked freezing remained low, and no sex differences were detected in either recent or remote retrieval (Figure 3A; two-way repeated-measures ANOVA, main effect of retrieval trial: F (3.609, 90.23) = 2.701, *p* = 0.041, ηp^2^ = 0.098; main effect of sex: F (1, 25) = 0.491, *p* = 0.490, ηp^2^ = 0.019; sex × retrieval trial interaction: F (9, 225) = 1.079, *p* = 0.379, ηp^2^ = 0.041; males = 13, females = 14). To further examine the possibility of spontaneous recovery at remote retrieval, we performed a planned comparison focused on Tone 1 of Day 13. Freezing during the first remote retrieval tone did not differ significantly between females and males (Figure 3A; Welch’s *t* test, t (19.27) = 0.409, *p* = 0.687, Hedges’ g = 0.16). In addition, the increase from late extinction to the first tone of remote retrieval, used here as an index of spontaneous recovery, did not differ significantly between sexes (Figure 3B; Welch’s *t* test, t (23.47) = 0.021, *p* = 0.983, Hedges’ g = 0.01). Thus, although the female group visually appeared to show a slightly greater rebound at Tone 1, this effect was not statistically reliable under the present experimental conditions. Freezing during recent retrieval tones (1–4) did not differ from freezing during the final day’s extinction tones (9–12). No sex differences were detected (Figure 3C; two-way repeated-measures ANOVA, main effect of session [Day 6 vs. Day 5]: F (1, 25) = 0.2040, *p* = 0.655, ηp^2^ = 0.008; main effect of sex: F (1, 25) = 0.1988, *p* = 0.660, ηp^2^ = 0.008; sex × session interaction: F (1, 25) = 0.069, *p* = 0.795, ηp^2^ = 0.003; post hoc comparisons, all adjusted *p* > 0.05, all |Hedges’ g| < 0.13; males = 13, females = 14). Likewise, freezing during remote retrieval tones 1–4 did not differ from freezing during the final day’s extinction tones 9–12, and no sex differences were detected (Figure 3D; two-way repeated-measures ANOVA, main effect of session [Day 13 vs. Day 5]: F (1, 25) = 0.2162, *p* = 0.646, ηp^2^ = 0.009; main effect of sex: F (1, 25) = 0.042, *p* = 0.839, ηp^2^ = 0.002; sex × session interaction: F (1, 25) = 1.232, *p* = 0.278, ηp^2^ = 0.047; post hoc comparisons, all adjusted *p* > 0.05, all |Hedges’ g| < 0.29; males = 13, females = 14). These results indicate that neither sex exhibited time-dependent enhancement of fear memory.

#### 3.1.2. Sex Does Not Significantly Influence Freezing Bout Counts or Locomotor Activity During Fear Extinction and Retrieval

**Figure 4:**
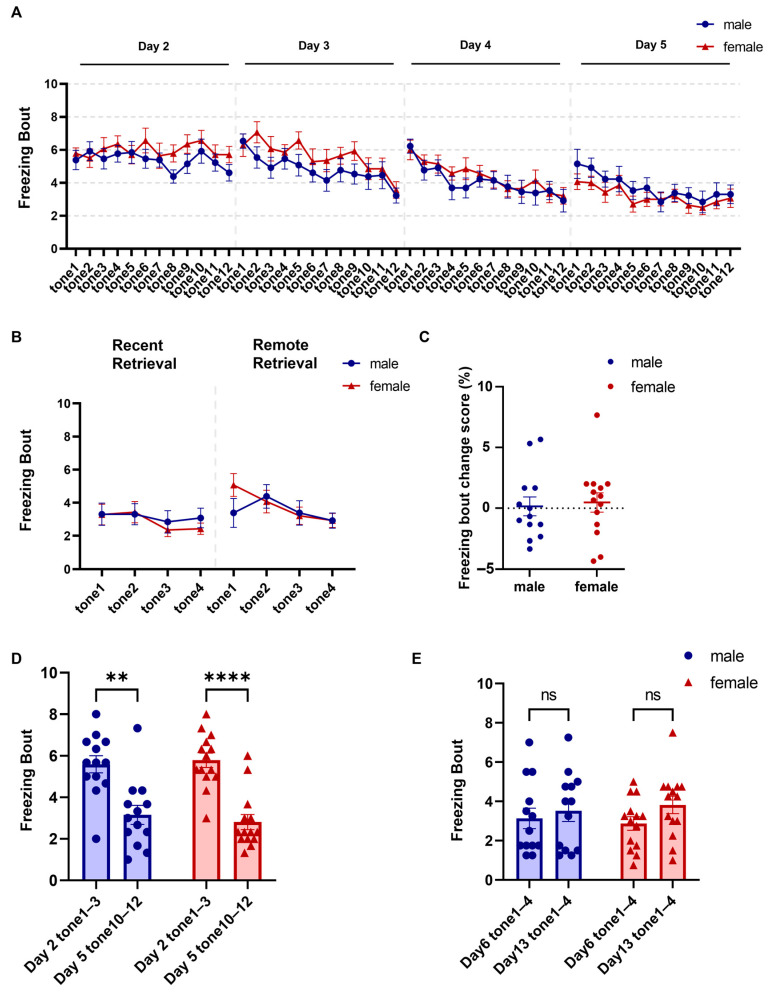
Comparable freezing bout counts in female and male mice during fear extinction and retrieval. (**A**) Freezing bout counts across fear extinction. (**B**,**E**) Freezing bout counts during fear retrieval. (**C**) Change in freezing bout count from late extinction to Tone 1 of remote retrieval in female and male mice. Each point represents one animal. Positive values indicate a higher freezing bout count during the first tone of remote retrieval than during late extinction, whereas negative values indicate no rebound or a lower freezing bout count relative to late extinction. (**D**) Freezing bout counts during early extinction (tones 1–3, Day 2) versus late extinction (tones 10–12, Day 5) in both sexes. Data are presented as mean ± SEM; *n* = 13 males and 14 females. Asterisks indicate statistically significant comparisons, **: *p* < 0.01; ****: *p* < 0.0001. Exact *p* values, test statistics, and effect sizes for the comparisons shown in this figure are reported in the Section 3.

The number of CS-evoked freezing bouts decreased progressively across extinction, with no significant sex differences (Figure 4A; two-way repeated-measures ANOVA, main effect of extinction trial: F (11.38, 284.5) = 7.999, *p* < 0.0001, ηp^2^ = 0.242; main effect of sex: F (1, 25) = 0.9776, *p* = 0.332, ηp^2^ = 0.038; sex × extinction trial interaction: F (47, 1175) = 0.7988, *p* = 0.833, ηp^2^ = 0.031; males = 13, females = 14). Freezing bouts during tones (1–3) on the first extinction day were significantly greater than during tones (10–12) on the last day, yet no sex difference was detected at either time point (Figure 4D; two-way repeated-measures ANOVA, main effect of extinction phase [Day 2 tones 1–3 vs. Day 5 tones 10–12]: F (1, 25) = 40.84, *p* < 0.0001, ηp^2^ = 0.620; main effect of sex: F (1, 25) = 0.042, *p* = 0.840, ηp^2^ = 0.002; sex × extinction-phase interaction: F (1, 25) = 0.4070, *p* = 0.529, ηp^2^ = 0.016; post hoc comparisons, male Day 2 tones 1–3 vs. Day 5 tones 10–12, adjusted *p* = 0.001, Hedges’ g = 1.04; female Day 2 tones 1–3 vs. Day 5 tones 10–12, adjusted *p* < 0.0001, Hedges’ g = 1.27; males = 13, females = 14).

**Figure 5:**
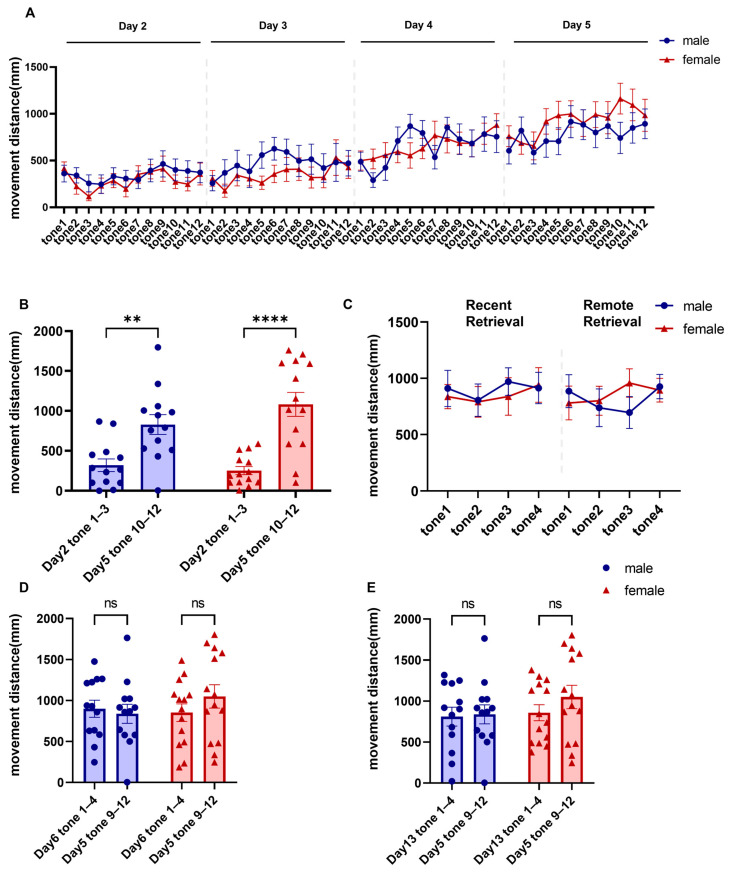
Comparable locomotor responses in female and male mice during fear extinction and retrieval. (**A**) Locomotor distance across fear extinction. (**B**) Locomotor distance during early versus late extinction in both sexes. (**C**) Locomotor distance during fear retrieval. (**D**) Locomotor distance during recent retrieval versus the final day of extinction. (**E**) Locomotor distance during remote retrieval versus the final day of extinction. Data are presented as mean ± SEM; *n* = 13 males and 14 females. Asterisks indicate statistically significant comparisons, **: *p* < 0.01; ****: *p* < 0.0001. Exact *p* values, test statistics, and effect sizes for the comparisons shown in this figure are reported in the Section 3.

As fear responses declined, locomotor distance in Context B increased. Consistent with the decline in freezing, locomotor distance during CS presentations increased across extinction days, further supporting successful fear extinction (Figure 5A; two-way repeated-measures ANOVA, main effect of extinction trial: F (11.33, 283.3) = 10.21, *p* < 0.0001, ηp^2^ = 0.290; main effect of sex: F (1, 25) = 0.006, *p* = 0.937, ηp^2^ = 0.0003; sex × extinction trial interaction: F (47, 1175) = 1.150, *p* = 0.228, ηp^2^ = 0.044; males = 13, females = 14). Distance traveled during tones (10–12) on the final extinction day was greater than during tones (1–3) on the first day, and no sex differences were found (Figure 5B; two-way repeated-measures ANOVA, main effect of extinction phase [Day 2 tones 1–3 vs. Day 5 tones 10–12]: F (1, 25) = 55.16, *p* < 0.0001, ηp^2^ = 0.688; main effect of sex: F (1, 25) = 0.5548, *p* = 0.463, ηp^2^ = 0.022; sex × extinction-phase interaction: F (1, 25) = 3.152, *p* = 0.088, ηp^2^ = 0.112; post hoc comparisons, male Day 2 tones 1–3 vs. Day 5 tones 10–12, adjusted *p* = 0.001, |Hedges’ g| = 1.02; female Day 2 tones 1–3 vs. Day 5 tones 10–12, adjusted *p* < 0.0001, |Hedges’ g| = 1.67; males = 13, females = 14).

During retrieval, freezing bout counts did not differ between recent and remote retrieval (Figure 4E; two-way repeated-measures ANOVA, main effect of retrieval time [Day 6 vs. Day 13]: F (1, 25) = 2.983, *p* = 0.097, ηp^2^ = 0.107; sex × retrieval-time interaction: F (1, 25) = 0.5314, *p* = 0.473, ηp^2^ = 0.021; post hoc comparisons, all adjusted *p* > 0.05, all |Hedges’ g| < 0.44; males = 13, females = 14), and no sex differences were found (Figure 4B; two-way repeated-measures ANOVA, main effect of sex: F (1, 25) = 0.002, *p* = 0.968, ηp^2^ < 0.001; sex × retrieval-time interaction: F (7, 175) = 0.9432, *p* = 0.475, ηp^2^ = 0.036; post hoc comparisons at each retrieval trial, all adjusted *p* > 0.05; males = 13, females = 14). We also examined freezing bout during Tone 1 of remote retrieval as an additional readout of spontaneous recovery. No significant sex difference was detected (Figure 4B; Welch’s *t* test, t (24.67) = 0.024, *p* = 0.981, Hedges’ g = 0.01). Likewise, the change in freezing bout from late extinction to the first remote retrieval tone did not differ significantly between sexes (Figure 4C; Welch’s *t* test, t (24.99) = 0.290, *p* = 0.774, Hedges’ g = 0.11). Locomotor distance also did not differ between sexes during either retrieval session (Figure 5C; two-way repeated-measures ANOVA, main effect of sex: F (1, 25) < 0.001, *p* = 0.997, ηp^2^ < 0.001; sex × retrieval-time interaction: F (7, 175) = 0.5179, *p* = 0.820, ηp^2^ = 0.02; post hoc comparisons at each retrieval trial, all adjusted *p* > 0.05; males = 13, females = 14). Furthermore, the distances traveled during tones (1–4) on Day 6 did not differ from those recorded during the final extinction day (Figure 5D; two-way repeated-measures ANOVA, main effect of session [Day 6 vs. Day 5]: F (1, 25) = 0.5225, *p* = 0.477, ηp^2^ = 0.021; main effect of sex: F (1, 25) = 0.3431, *p* = 0.563, ηp^2^ = 0.014; sex × session interaction: F (1, 25) = 1.880, *p* = 0.183, ηp^2^ = 0.07; post hoc comparisons, all adjusted *p* > 0.05, all |Hedges’ g| < 0.38; males = 13, females = 14). Likewise, the distances traveled during tones (1–4) on Day 13 did not differ from those recorded during the final extinction day (Figure 5E; two-way repeated-measures ANOVA, main effect of session [Day 13 vs. Day 5]: F (1, 25) = 1.793, *p* = 0.193, ηp^2^ = 0.067; main effect of sex: F (1, 25) = 0.7694, *p* = 0.389, ηp^2^ = 0.030; sex × session interaction: F (1, 25) = 1.015, *p* = 0.323, ηp^2^ = 0.039; post hoc comparisons, all adjusted *p* > 0.05, all |Hedges’ g| < 0.42; males = 13, females = 14).

Because footshocks were administered during fear acquisition, locomotor distance was not quantified during that phase.

In summary, during conditioned fear extinction and retrieval, fear levels and locomotor activity toward the conditioned stimulus were not influenced by sex.

### 3.2. Safety Conditioning

**Figure 6:**
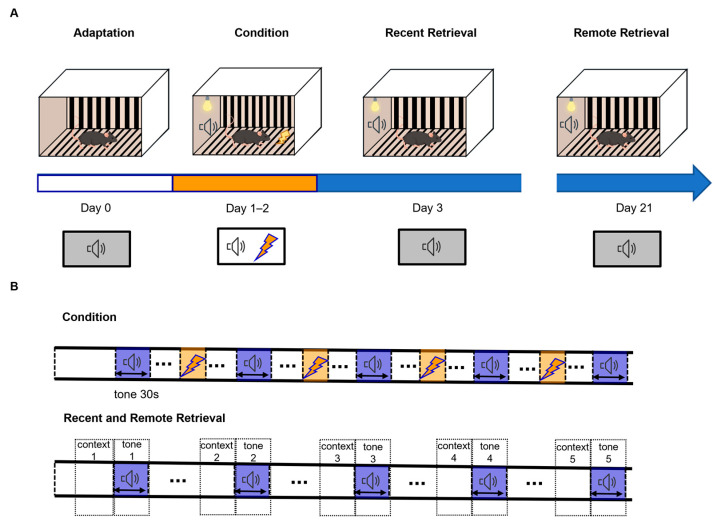
Schematic for the safety conditioning procedure. (**A**) Experimental context and procedure. (**B**) Timing of conditioned stimulus presentation and intertrial interval.

A previously established safety-conditioning paradigm was employed [17,42,43] wherein mice received footshocks at random times within the training context, but never during auditory cue presentations, establishing explicit safety periods. Both male and female mice underwent a recent retrieval test one day after conditioning and a remote retrieval test 18 days later (Figure 6A,B). Each retrieval session included five CS trials. Contextual freezing was recorded during the 30 s preceding each CS presentation, while CS-evoked freezing was measured during each CS (Figure 6A,B).

Sex Does Not Affect Baseline, Contextual, or CS-Period Freezing in C57BL/6J Wild-Type Mice During Recent and Remote Safety Memory Retrieval

**Figure 7:**
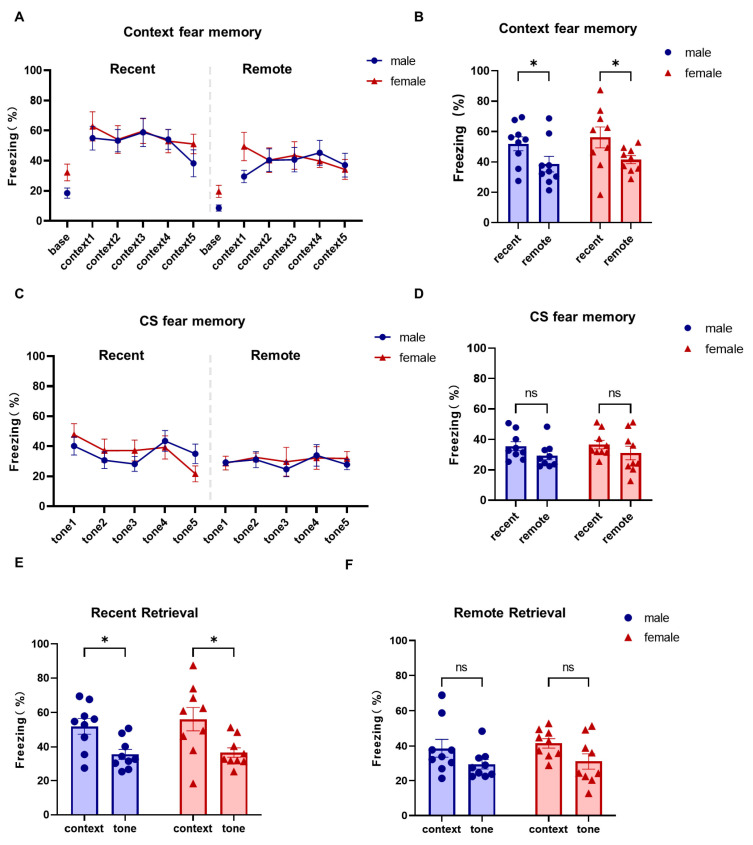
Comparable baseline, contextual, and CS-period freezing during recent and remote safety retrieval in female and male mice. (**A**) Baseline freezing (0–180 s) and trial-by-trial contextual freezing during recent and remote retrieval in female and male mice. Contextual freezing was measured during the 30 s preceding each CS presentation. (**B**) Mean contextual freezing during recent and remote retrieval in both sexes. (**C**) Trial-by-trial CS-period freezing during recent and remote retrieval in female and male mice. (**D**) Mean CS-period freezing during recent and remote retrieval in both sexes. (**E**) Comparison of mean contextual freezing and mean CS-period freezing during recent retrieval in both sexes. (**F**) Comparison of mean contextual freezing and mean CS-period freezing during remote retrieval in both sexes. Data are presented as mean ± SEM; *n* = 9 mice per sex. Asterisks indicate statistically significant comparisons. Exact *p* values, test statistics, and effect sizes for the comparisons shown in this figure are reported in the Section 3.

During the process of safe memory retrieval, there was no significant difference in the baseline freezing behavior between female and male mice during the initial 0–180 s period, indicating that there was no sex difference in the fear levels before the presentation of the conditioned stimulus. Trial-by-trial contextual freezing during retrieval also did not differ significantly between sexes in either the recent or remote test (Figure 7A; two-way repeated-measures ANOVA, main effect of sex: F (1, 16) = 0.7824, *p* = 0.390, ηp^2^ = 0.191; main effect of retrieval time [recent vs. remote]: F (6.230, 99.69) = 8.524, *p* < 0.0001, ηp^2^ = 0.170; sex × retrieval-time interaction: F (11, 176) = 0.7392, *p* = 0.700, ηp^2^ = 0.047; males = 9, females = 9). When the mean contextual freezing values were compared, contextual freezing was significantly lower during remote retrieval than during recent retrieval in both sexes (Figure 7B; two-way repeated-measures ANOVA, main effect of retrieval time: F (1, 16) = 15.96, *p* = 0.001, ηp^2^ = 0.499; main effect of sex: F (1, 16) = 0.3274, *p* = 0.575, ηp^2^ = 0.020; sex × retrieval-time interaction: F (1, 16) = 0.03739, *p* = 0.849, ηp^2^ = 0.002; post hoc comparisons, male recent vs. remote retrieval, adjusted *p* = 0.032, Hedges’ g = 0.81; female recent vs. remote retrieval, adjusted *p* = 0.018, Hedges’ g = 0.89; males = 9, females = 9). These findings indicate that contextual freezing decreased from recent to remote retrieval in both male and female mice.

CS-period freezing likewise showed no significant sex differences during either recent or remote retrieval (Figure 7C; two-way repeated-measures ANOVA, main effect of sex: F (1, 16) = 3.779, *p* = 0.070, ηp^2^ = 0.191; main effect of retrieval time [recent vs. remote]: F (4.561, 72.98) = 3.280, *p* = 0.012, ηp^2^ = 0.170; sex × retrieval-time interaction: F (11, 176) = 0.7871, *p* = 0.653, ηp^2^ = 0.047; males = 9, females = 9). Furthermore, in both sexes, there was no significant difference in the mean CS-period freezing for the recent and remote retrieval (Figure 7D; two-way repeated-measures ANOVA, main effect of retrieval time: F (1, 16) = 2.163, *p* = 0.161, ηp^2^ = 0.119; main effect of sex: F (1, 16) = 0.2838, *p* = 0.601, ηp^2^ = 0.017; sex × retrieval-time interaction: F (1, 16) = 0.005, *p* = 0.943, ηp^2^ < 0.001; post hoc comparisons, male recent vs. remote retrieval, adjusted *p* = 0.498, Hedges’ g = 0.33; female recent vs. remote retrieval, adjusted *p* = 0.562, Hedges’ g = 0.30; males = 9, females = 9). These results indicate that cue-related freezing remained stable across the two retrieval time points. During recent retrieval, mean contextual freezing was significantly higher than mean CS-period freezing in both sexes (Figure 7E; two-way repeated-measures ANOVA, main effect of freezing type [contextual vs. CS-period]: F (1, 16) = 15.19, *p* = 0.001, ηp^2^ = 0.487; main effect of sex: F (1, 16) = 0.3271, *p* = 0.575, ηp^2^ = 0.020; sex × freezing-type interaction: F (1, 16) = 0.1173, *p* = 0.737, ηp^2^ = 0.007; post hoc comparisons, male contextual vs. CS-period freezing, adjusted *p* = 0.046, Hedges’ g = 0.76; female contextual vs. CS-period freezing, adjusted *p* = 0.017, Hedges’ g = 0.90; males = 9, females = 9). In contrast, during remote retrieval, contextual and CS-period freezing did not differ significantly in either sex (Figure 7F; two-way repeated-measures ANOVA, main effect of freezing type [contextual vs. CS-period]: F (1, 16) = 5.220, *p* = 0.563, ηp^2^ = 0.246; main effect of sex: F (1, 16) = 0.4272, *p* = 0.523, ηp^2^ = 0.026; sex × freezing-type interaction: F (1, 16) = 0.020, *p* = 0.889, ηp^2^ = 0.001; post hoc comparisons, male contextual vs. CS-period freezing, adjusted *p* = 0.276, Hedges’ g = 0.46; female contextual vs. CS-period freezing, adjusted *p* = 0.200, Hedges’ g = 0.52; males = 9, females = 9). Overall, these results indicate that during safety-memory retrieval, female and male mice exhibited comparable baseline, contextual, and CS-period freezing. The context–CS difference observed during recent retrieval was attenuated at the remote test, primarily because contextual freezing declined over time.

## 4. Discussion

The present study demonstrates that female and male C57BL/6J mice performed similarly across multiple domains of fear regulation under standardized experimental conditions. In the auditory fear-conditioning paradigm, both sexes acquired conditioned fear to a comparable extent, showed progressive extinction across sessions, and exhibited similarly low freezing during both recent and remote retrieval. In the safety-conditioning paradigm, both females and males displayed reduced freezing during the safety cue relative to contextual baseline, and contextual freezing further declined from recent to remote retrieval. Importantly, this convergence was observed across multiple behavioral indices, including freezing percentage, freezing-bout counts, and locomotor distance, indicating that the absence of sex effects was not limited to a single measure of conditioned responding. Together, these findings suggest that, under the present task parameters, sex was not a major determinant of behavioral outcome in auditory fear extinction or safety learning.

A likely explanation for the absence of significant sex differences in the present study is that the experimental design reduced several factors that often amplify sex-dependent behavioral divergence. In the fear-extinction paradigm, female and male mice entered extinction and retrieval from comparable acquisition levels, reducing the possibility that later differences simply reflected unequal initial fear learning. In addition, extinction and retrieval were assessed under standardized auditory conditions in a context distinct from conditioning, which may have reduced contextual and stress-related variability. In the safety-learning paradigm, the explicitly unpaired cue–shock design provided a relatively constrained assay of conditioned inhibition, under which sex-related divergence may be less pronounced. Moreover, the absence of sex effects was replicated across multiple behavioral indices, including freezing percentage, freezing-bout counts, and locomotor distance, arguing against the possibility that the null result arose solely from insensitivity of a single behavioral readout.

These findings are broadly consistent with previous rodent studies showing that sex differences in fear acquisition are often modest or absent [23,44,45,46,47,48]. However, the literature on fear extinction is more mixed. In rats, several studies have reported that males show higher freezing during extinction or that females extinguish fear more rapidly [23,45,46,49], whereas mouse studies, particularly in C57BL/6J animals, have yielded more variable or strain-dependent outcomes [44,50]. One explanation for this discrepancy is that sex effects in fear regulation are highly sensitive to species and strain background. Rats and mice differ in stress responsivity, locomotor tendencies, defensive repertoire, and baseline emotionality, any of which may influence how extinction learning is expressed behaviorally. In addition, strain-specific neural and endocrine profiles may shape the degree to which gonadal hormones modulate fear-related behavior, making direct comparisons across species and strains difficult.

Notably, our findings are also consistent with previous reports that sex differences become more apparent when estrous phase is explicitly monitored or experimentally controlled, whereas studies allowing animals to remain in natural hormonal states often report reduced or inconsistent sex effects [50,51]. Accordingly, the present data should not be interpreted as evidence that estrous-cycle effects were absent. Rather, they indicate that any phase-specific hormonal influences were not sufficiently large or systematic to generate robust group-level behavioral differences under the standardized conditions used here, particularly when females were analyzed as a naturally cycling group rather than stratified by cycle stage.

Hormonal mechanisms nevertheless remain highly relevant to the interpretation of these findings. Estradiol and progesterone are known to influence fear extinction and fear-memory processing through actions on the amygdala, hippocampus, and medial prefrontal cortex, as well as through modulation of dopaminergic, serotonergic, endocannabinoid, and BDNF-related signaling pathways [29,30,52,53]. Testosterone may also influence stress reactivity and fear memory through effects on limbic circuits and the hypothalamic–pituitary–adrenal axis [29,30,54,55]. One possibility is that the behavioral effects of hormonal fluctuations are state-dependent and emerge only under conditions of heightened stress, greater cognitive demand, or more ambiguous cue contingencies than those used here. Another possibility is that males are not hormonally static controls; rather, fluctuations in testosterone, social dominance, or stress responsivity may generate behavioral variability that parallels endocrine-related variability in females, thereby masking apparent group-level sex differences [56,57]. From this perspective, the current null result should not be taken to mean that sex hormones are behaviorally irrelevant, but instead that endogenous hormonal variation in both sexes did not translate into robust between-sex differences under the present testing conditions. Future studies combining hormonal assays with pharmacological or endocrine manipulations will be important for disentangling these possibilities.

Several limitations should be acknowledged. First, the estrous cycle was not monitored, and therefore phase-specific hormonal effects cannot be excluded. Because females were analyzed as a naturally cycling group, any estrous-dependent variation may have been averaged across animals and thus may not have emerged as a robust between-sex behavioral difference. This is an important limitation, given evidence that extinction performance can vary across estrous stages when hormonal status is explicitly assessed [51]. Second, although the present sample sizes were sufficient to detect robust learning effects, they may still have been underpowered to detect subtle sex-by-time interactions, especially in the safety-conditioning cohort. Therefore, the absence of significant sex differences in the present study should be interpreted cautiously as evidence of comparable performance under the current experimental conditions, rather than as definitive evidence that sex has no effect on fear extinction or safety learning. Third, no no-extinction control group was included in the present study. Therefore, although freezing decreased across extinction sessions and remained low during retrieval, we cannot conclude unequivocally that these retrieval effects exclusively reflect the effectiveness or retention of extinction training, as other factors such as repeated cue exposure or time-dependent attenuation of conditioned responding may also have contributed. Fourth, locomotor distance was not analyzed during fear acquisition because footshock delivery elicited abrupt unconditioned reactions in some mice, including jumping-like escape responses, making distance traveled during this phase difficult to interpret as a stable measure of locomotor behavior. Under these conditions, movement during acquisition would primarily reflect immediate shock reactivity rather than locomotor activity directly comparable to that measured during extinction or retrieval. Accordingly, although our later locomotor analyses showed no sex differences, we cannot exclude the possibility of subtle sex differences in active shock responses during acquisition because these were not quantified separately. Finally, the present study examined a single strain, age range, and task configuration; therefore, the conclusion of no sex difference should be interpreted as context-specific rather than universally generalizable. In other words, our findings support behavioral equivalence under these experimental conditions, but they do not exclude the possibility that sex-dependent effects may emerge in other paradigms, under chronic stress, or when hormonal state is directly assessed. It also remains unclear whether the same pattern would be observed in appetitive learning or appetitive extinction paradigms. This question is important because appetitive and aversive cue updating relies on partially overlapping, but not identical, neural and motivational processes [58]. In addition, prior rodent studies suggest that sex differences may emerge in some appetitive tasks, including food-cue renewal and reward-related conditioned inhibition [59,60]. Accordingly, direct comparison of sex effects across aversive and appetitive paradigms will be an important direction for future work.

From a translational perspective, the present findings are important because both fear extinction and safety learning model adaptive inhibitory processes that are highly relevant to anxiety- and trauma-related disorders and closely parallel mechanisms engaged by exposure-based treatments for PTSD [12,15,18,19]. Our observation that female and male mice showed comparable performance across these paradigms supports the inclusion of both sexes in preclinical studies of fear regulation, particularly in experiments not specifically designed to test sex as the primary independent variable. This interpretation is further supported by meta-analytic evidence indicating that females are not inherently more variable than males across neuroscience and fear-related behavioral datasets [36,61,62,63]. At the same time, the absence of sex differences in the present paradigms appears to contrast with clinical epidemiology showing a consistently higher lifetime prevalence of PTSD in women [22,23,26,29]. This discrepancy suggests that factors beyond basic fear inhibition may contribute to sex disparities in humans, including differences in trauma exposure, cognitive appraisal, coping style, endocrine state, and broader social determinants of mental health [21,22,23]. Alternatively, hormonal influences on fear regulation may emerge more strongly during fear memory consolidation, reconsolidation, or under chronic or social stress, rather than during standard extinction procedures alone [30,52,55]. Future work should therefore move beyond asking whether the sexes differ on average and instead investigate when sex-dependent effects emerge, under what hormonal or stress-related states they become detectable, and through which neural or environmental pathways they acquire behavioral and clinical significance. Integrating endocrine monitoring, social stressors, trauma-relevant environmental manipulations, and longitudinal designs may be especially useful for bridging the gap between controlled preclinical findings and the more complex sex disparities observed in human PTSD.

## 5. Conclusions

In conclusion, using standardized auditory fear-conditioning, extinction, retrieval, and safety-learning paradigms, we found no evidence that sex systematically altered freezing or locomotor behavior in C57BL/6J mice. Both female and male mice acquired conditioned fear, showed progressive reductions in freezing across extinction sessions, and exhibited similarly low freezing during recent and remote retrieval tests under matched experimental conditions. They also showed comparable behavioral performance in the safety-learning paradigm. The significance of this result lies not simply in the absence of a sex difference, but in the consistency of that absence across multiple behavioral indices and across two complementary fear-regulation paradigms. These data argue against the assumption that female mice are intrinsically more behaviorally variable or less suitable for preclinical fear research. At the same time, they do not rule out context-dependent hormonal modulation; rather, they suggest that such modulation may be subtle, conditional, or shared across sexes. Accordingly, sex should be treated as an essential biological variable in study design and interpretation, but not as a justification for excluding females from preclinical research on fear regulation [28,32].

## Data Availability

The original contributions presented in this study are included in the article. Further inquiries can be directed to the corresponding author.
